# Coagulation Abnormalities and Risk Assessment in Acute Promyelocytic Leukemia: An Experience From a Resource-Constraint Country

**DOI:** 10.7759/cureus.26026

**Published:** 2022-06-17

**Authors:** Warkha Thakur, Nida Anwar, Naveena Fatima, Aisha Jamal, Quratul Ain Rizvi, Munira Borhany

**Affiliations:** 1 Department of Hematology, National Institute of Blood Diseases and Bone Marrow Transplantation, Karachi, PAK; 2 Department of Hematology and Bone Marrow Transplant, National Institute of Blood Diseases and Bone Marrow Transplantation, Karachi, PAK; 3 Department of Research and Development, National Institute of Blood Diseases and Bone Marrow Transplantation, Karachi, PAK

**Keywords:** pakistan, fibrinolysis, coagulopathy, disseminated intravascular coagulation, apml

## Abstract

Introduction

The objective of the study was to assess the impact of coagulopathy in risk-stratified acute promyelocytic leukemia (APML) patients irrespective of bleeding manifestation.

Patients and methods

This was a cross-sectional study design conducted at the National Institute of Blood Diseases and Bone Marrow Transplantation (NIBD & BMT) from November 2019 to December 2021. A total of 62 patients between three years to 74 years of age of either gender and treatment-naive cases of APML were included in the study. Morphological diagnosis was made on bone marrow samples, and confirmation was done by karyotyping/fluorescence in situ hybridization (FISH) and/or polymerase chain reaction (PCR). Complete blood count (CBC), prothrombin time (PT), activated partial thromboplastin time (APTT), D-dimer, and fibrinogen levels were done for bleeding risk assessment. Cases other than APML and cases on treatment were excluded from the study.

Results

A total of 85 APML patients were registered at our institute. Among them, 62 (73%) were included in the analysis as per the inclusion criteria of the study. The median age was 32 (3-74) years, with a male predominance of 34 (55%). According to the Sanz score, 18 (29%) patients were noted to have low risk; however, 22 (35.4%) patients were found to have an intermediate-risk disease and 22 (35.4%) patients had high-risk disease. There was positive bleeding history among 44 (71%) patients, followed by fever in 28 (45%) patients. Raised PT, APTT, and D-dimer were found in 46 (74%), 38 (61%), and 52(83.8%) patients, respectively. Low fibrinogen levels were observed among 16 (26%) patients. The association of risk stratification and bleeding history with CBC and coagulation parameters was observed. Platelet count and total leucocyte count were noted to be significantly associated with risk stratification. However, there was no association observed between the rest of the parameters with risk stratification and bleeding.

Conclusion

The results of our study suggest that regardless of bleeding symptoms, coagulation parameters must be investigated at the time of diagnosis in patients with suspected APML, and in addition to all-trans-retinoic acid (ATRA), transfusion of fresh frozen plasma should be done. It has clinical value, and adding it to the algorithm of treatment would be beneficial to the patients in the developing world, where resources are already meager.

## Introduction

Acute myeloid leukemia (AML) is a clonal hematological disorder resulting from maturation abnormalities of the myeloid lineage. Acute promyelocytic leukemia (APML) is the M3 subtype of AML according to the French-American-British (FAB) classification [1]. LK Hillestad, a Norwegian hematologist, was the first person to describe and report APML in 1957. After that, J Bernard in 1959 more extensively described the disease with its connection to promyelocytic proliferation while reporting 20 patients diagnosed with APML [2]. It results from a reciprocal translocation between chromosome 15 and 17 retinoic acid receptor alpha (RARα), leading to fusion gene formation PML-RARA and clonal proliferation of abnormal promyelocytes [3]. APML was once considered the most malignant form of leukemia; however, treatment outcomes have improved tremendously with the advent of all-trans-retinoic acid (ATRA) and arsenic trioxide (ATO), achieving remarkable cure rates. However, there is an increased risk of hemorrhagic and thrombotic complications. Platelet, thrombomodulin, and protein C receptor expression contribute to these implications [4]. Rapid diagnosis of the disease and early assessment of risk is required so that serious complications can be averted, thus, in turn, reducing morbidity and mortality.

Furthermore, data on complete coagulation profiles and parameters in developing countries such as Pakistan is either unavailable or very limited. Notwithstanding the various uncertainties, the literature nevertheless stresses the significance of measures that provide supportive care to APML patients who have abnormalities of coagulation along with receiving chemotherapy [5].

One of the main features of APML is the existence of abnormalities in coagulation pathways, due to which around 60% of the cases present with minor bleeding at the time of diagnosis. Prior to the availability of ATRA, early death due to hemorrhagic events was manifested in 10%-30% of the patients [6]. Despite the higher rates of cure that are described, coagulopathy remains to be the primary cause accountable for mortality and one of the reasons for early morbidity in the course of the disease [5].

The clinical profile of APML patients at the time of presentation includes prolonged activated partial thromboplastin time (APTT), prothrombin time (PT), high D-dimers, decreased platelet counts, and hypofibrinogenemia. The fibrinopeptide A and thrombin-antithrombin complex (TAT) are also increased. This depicts thrombin formation intravascularly. It also represents stimulation of the coagulation cascade in vivo, signifying that a method analogous to disseminated intravascular coagulation (DIC) might exist [7]. Moreover, APML blast cells release tissue factor (TF) and produce interleukin, stimulating the cascade of coagulation. One of the other procoagulants called cancer procoagulant, which stimulates Factor X by itself, is existent in the cells of APML. More recently, it has also been reported that the expression of CD44 on the membrane of APML blast cells was related to increased binding of fibrinogen, leading to an abnormal fibrin distribution [8]. In addition, there are some particularities that are noted in APML coagulopathy. For instance, the platelet half-life and plasma concentrations of protein C and antithrombin are normal, in turn depicting a different picture from classical DIC. The disparity between laboratory data and clinical bleeding indicates that different mechanisms might also be a causative factor(s) [5,9].

APML patients are typically classified into non-high-risk and high-risk patients based on WBC counts according to the European LeukemiaNet (ELN) recommendations. Patients are considered non-high-risk when WBC counts are ≤10 × 10^9^/L, while patients having WBC counts >10 × 10^9^/L are categorized as high-risk. Treatment options differ for both groups. For non-high-risk patients, treatment with the combination of ATRA and ATO without cytoreductive chemotherapy is strongly supported by randomized clinical trials and hence recommended as the standard of care. For high-risk patients, there are two treatment options with neither being proven superior to the other in clinical trials. High-risk patients can be treated with the combination of ATRA, ATO, and cytoreductive chemotherapy or ATRA plus chemotherapy [10].

Hence, while treating APML patients, prompt ATRA administration is of paramount significance, which has been shown to improve outcomes and revert coagulopathy to a substantial extent. In addition, the platelet counts should be kept above 20-30 × 10^6^/μL via transfusion, and transfusion of fresh frozen plasma (FFP) and cryoprecipitate (CP) should be done to keep fibrinogen above 1-1.5 g/L. Antifibrinolytic drug administration may also improve the clinical outcome. Due to worsening bleeding diathesis, leukocytapheresis should be avoided [11].

Developing countries such as Pakistan have unavailable or very limited data on complete coagulation profiles and parameters. Moreover, routine screening of coagulation parameters is scarcely done. The objective of the study was to assess the impact of coagulopathy in risk-stratified APML patients presenting with or without bleeding diathesis.

## Materials and methods

This was a cross-sectional study conducted at the National Institute of Blood Diseases and Bone Marrow Transplantation (NIBD & BMT) from November 2019 to December 2021. Approval from the institutional ethics review committee (ERC) was taken prior to conducting the study.

All treatment-naive cases of APML were included in the study. Cases other than APML and cases on treatment were excluded. The demographic and clinical features including age, gender, and presence of bleeding events were recorded. The Sanz score was used for risk stratification. The diagnosis was done morphologically by doing bone marrow biopsies followed by confirmation by karyotyping and detection of PML-RARA fusions by either fluorescence in situ hybridization (FISH) or polymerase chain reaction (PCR). Laboratory tests to assess the bleeding risk include complete blood count (CBC), PT, APTT, D-dimer, and fibrinogen level. Sysmex XN-1000 analyzer (Sysmex Corporation, Kobe, Japan) was used to obtain the CBC including platelet counts. An automated latex-enhanced immunoassay method was used to assess the D-dimer levels. Fibrinogen levels were obtained using the Clauss fibrinogen assay method. PT and APTT were performed on STAGO and Sysmex CA-1500 through the immunoturbidimetric method. Peripheral smear and bone marrow aspirates were stained by Leishman’s stain and analyzed by a hematologist. Chromosome analysis required five principal steps: (1) cell culture, (2) harvest of metaphase chromosomes, (3) chromosome preparation, (4) banding and staining using Giemsa and trypsin, and (5) analysis by light microscopy or karyotype-assisted computer analysis [4]. The addition of colchicine (or colcemid) pretreatment results in a mitotic arrest, and the treatment of arrested cells with a hypotonic solution such as potassium chloride improved the yield and quality of metaphase spreads. FISH included cell culture, harvesting of chromosomes at the interphase stage, slide preparation with probe application, denaturation of the sample and probe at 75c, hybridization at 37c, post-hybridization washes by saline-sodium citrate (SSC), and counterstain with 4',6-diamidino-2-phenylindole (DAPI).

Data were analyzed using the Statistical Package for the Social Sciences (SPSS) version 23.0 (IBM Corporation, Armonk, NY, USA). The Shapiro-Wilk test was applied, and data were normally distributed. For qualitative variables, frequency and percentages were calculated, while mean and standard deviation were computed for quantitative variables. The chi-square test was applied to observe the association between categorical variables. ANOVA was applied to observe the mean difference of laboratory parameters between the risk stratification groups, and multiple comparison was performed by applying the least significant difference (LSD). Survival analysis was done using Kaplan-Meier. P-value < 0.05 was considered to be statistically significant.

## Results

A total of 85 APML patients were registered at our institute during the study duration. Out of the total, 62 (73%) patients were analyzed as per our study’s inclusion criteria. The mean age was found to be 32 ± 15.8 years, with a male predominance of 34 (55%). The results of the baseline investigations are given in Table 1.

**Table 1:** Baseline Laboratory Parameters

Characteristic	Mean ± standard deviation
Age (years)	32 ± 15.8
Prothrombin time (seconds)	17.7 ± 4.9
Activated partial thromboplastin time (seconds)	32 ± 6.4
D-dimer (µg/mL)	6.1 ± 13.9
Fibrinogen (g/L)	2.7 ± 1.3
Hemoglobin (g/dL)	9.2 ± 1.8
Red blood cell count (×10^12^/L)	3.1 ± 0.7
Absolute retic count (×10^9^/L)	2.4 ± 1.5
Packed cell volume (%)	27 ± 5.9
Mean corpuscular volume (fl)	89.6 ± 8.8
Mean corpuscular hemoglobin (pg)	29.5 ± 3.3
Mean corpuscular hemoglobin concentration (g/dL)	33 ± 2.5
Total leukocyte count (×10^9^/L)	12.5 ± 6.4
Platelets (×10^9^/L)	119 ± 114

As per the Sanz score, 18 (29%) patients were found to have a low risk and 22 (35.4%) patients were found to have an intermediate risk, and similarly, 22 (35.4%) patients were at high risk. A history of bleeding was found in 44 (71%) of the patients, followed by fever noted among 28 (45%) patients. The most frequent bleeding symptom in males was gum bleeding in 18 out of 34 (53%), and menorrhagia was noted in six out of 26 (23%) reproductive female patients. Raised PT, APTT, and D-dimer were observed in 46 (74.2%), 38 (61.3%), and 52 (83.8%) patients, respectively. Low fibrinogen levels were found in 16 (26%) patients. The mean difference of CBC parameters and age in low-, intermediate-, and high-risk groups was observed, and it is presented in Table 2. It was found that platelet, TLC, and retic count were statistically significant in one of the groups, with P-values of 0.001, 0.000, and 0.022, respectively.

**Table 2:** Association of Age and CBC Parameters With Risk Stratification *The mean difference is significant at the 0.05 level.

Variable	Risk stratification		P-value	95% confidence interval	
				Lower bound	Upper bound
Age (years)	Low	Intermediate	0.704	-10.97	16.04
	Intermediate	High	0.807	-11.27	14.36
	High	Low	0.541	-17.59	9.43
Hemoglobin (g/dL)	Low	Intermediate	0.278	-0.78	2.62
	Intermediate	High	0.991	-1.61	1.62
	High	Low	0.273	-2.63	0.77
Red blood cell count (×10^12^/L)	Low	Intermediate	0.411	-0.43	1.03
	Intermediate	High	0.825	-0.77	0.62
	High	Low	0.539	-0.95	0.51
Packed cell volume (%)	Low	Intermediate	0.143	-1.44	9.52
	Intermediate	High	0.79	-5.88	4.52
	High	Low	0.22	-8.83	2.13
Mean corpuscular hemoglobin (pg)	Low	Intermediate	0.761	-2.72	3.68
	Intermediate	High	0.738	-2.54	3.54
	High	Low	0.535	-4.18	2.22
Mean corpuscular hemoglobin concentration (g/dL)	Low	Intermediate	0.448	-3.29	1.49
	Intermediate	High	0.522	-1.55	2.99
	High	Low	0.878	-2.21	2.57
Platelets (×10^9^/L)	Low	Intermediate	0.000*	138.31	360.3
	Intermediate	High	0.337	-155.48	55.12
	High	Low	0.001*	-310.12	-88.13
Total leukocyte count (×10^9^/L)	Low	Intermediate	0.826	-7.2	8.95
	Intermediate	High	0.000*	-30.39	-15.08
	High	Low	0.000*	13.79	29.93
Absolute retic count (×10^9^/L)	Low	Intermediate	0.135	-0.67	4.74
	Intermediate	High	0.354	-1.38	3.75
	High	Low	0.022*	-5.92	-0.51
Mean corpuscular volume (fl)	Low	Intermediate	0.409	-4.8	11.44
	Intermediate	High	0.908	-8.14	7.27
	High	Low	0.473	-11	5.23

The association of risk stratification and bleeding status was evaluated with coagulation parameters, and no statistically significant association was found (Table 3).

**Table 3:** Association of Coagulation Parameters With Risk Stratification and Bleeding Status

Coagulation parameter		Risk stratification			P-value	Bleeding		P-value
		Low	Intermediate	High		Yes	No	
Raised prothrombin time (seconds)	No	8	4	4	0.317	12	4	0.771
	Yes	10	18	18		32	14	
Raised activated partial thromboplastin time (seconds)	No	10	4	10	0.198	18	6	0.694
	Yes	8	18	12		26	12	
Raised D-dimer (µg/mL)	Yes	16	18	18	0.889	38	14	0.555
	No	2	4	4		6	4	
Fibrinogen level (g/L)	High	2	4	4	0.837	8	2	0.809
	Low	4	4	8		12	4	
	Normal	12	14	10		24	12	
Platelet (×10^9^/L)	≥50	-	-	-	-	12	10	0.135
	<50	-	-	-	-	32	8	

**Figure 1:**
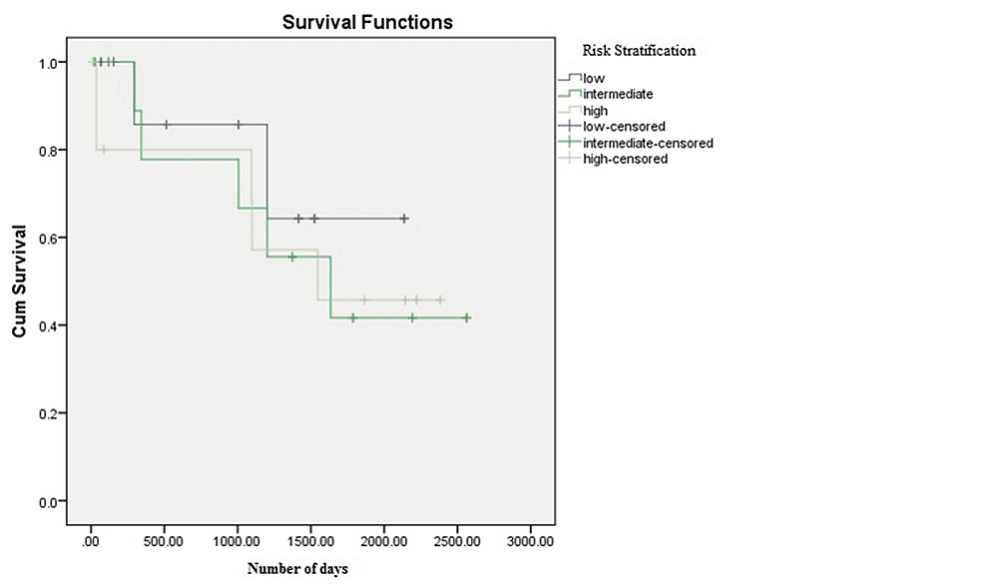
Kaplan-Meier Curve Survival Analysis Among Risk Groups

The total mean days of follow-up in the low-, intermediate-, and high-risk groups were 923 ± 690, 1140 ± 845, and 1138 ± 937 days, respectively. Out of 62, 24 (39%) patients were expired, and the overall survival rate was 61%. The percentage of alive and expired patients was found to be statistically nonsignificant among the low-, intermediate-, and high-risk groups (P = 0.234). The survival analysis with respect to risk stratification is shown in Figure 1.

## Discussion

This cross-sectional study was conducted to assess the impact of coagulopathy in risk-stratified APML patients. A total of 62 patients were analyzed. The mean age was 32 years. As per the Sanz score, 18 (29%) were designated as low-risk, 22 (35.5%) as intermediate-risk, and 22 (35.5%) patients as high-risk. Out of the 62 patients, 24 (39%) patients succumbed, and the overall survival rate was 61%. These results are comparable to the retrospective study carried out in India by Yedla et al. spanning a decade, in which the median age was reported to be 33 years [9]. In that study, the risk stratification revealed 43.3% of patients as high-risk, 41.4% of patients as intermediate-risk, and 15.3% as low-risk, and an overall survival rate of 74.4% was reported. Similarly, in a 12-year study by Steffenello-Durigon et al. in Brazil, the mean age was 34 years. Of the patients, 15.9% were categorized as low-risk, 59.1% as intermediate-risk, and 25% as high-risk, with an overall survival rate of 82.7% [12]. Abaza et al. reported a mean age of 50 years with 29% high-risk patients and 71% low-risk patients and an overall survival rate of 88% [13]. Ahmed et al. reported a survival rate of 37.8% [14], while Shein et al. reported a 76.5% survival rate [15].

Bleeding was reported in a total of 44 (71%) of the patients at presentation. The mean hemoglobin was 9.20 g/dL. The mean WBC count was 12.5 × 10^9^/L, while the mean platelet count was 119 × 10^9^/L. Similar results were reported by Ahmed et al. in Iraq, having 64.4% of patients presenting with bleeding, with mean hemoglobin of 8.31 g/dL, yet a mean WBC count of 22.3 × 10^9^/L; the mean platelet count was reported to be 33.37 × 10^9^/L [14]. Shah et al. in their study reported that the mean value of the WBC count at diagnosis was 42.3 × 10^3^/mm^3^, while the mean platelet count was 28 × 10^3^/mm^3^ [16]. Shein et al. in their study from South Africa reported coagulopathy in 60% of the patients at the time of diagnosis [15]. In contrast, Bewersdorf et al. reported a lower number of patients presenting with coagulopathy at 36.2% [17].

D-Dimers were found to be raised in 52 (83.8%) of the patients, with a mean value of 6.1 µg/mL, of which 38 patients presented with bleeding. The results are comparable to the study of Wang et al. in China [18]. The laboratory finding also concurs with the results of the retrospective cohort by Shahmarvand et al., in which they regarded raised levels of D-dimer as a sensitive indicator of APML [19]. In our study, raised levels of D-dimer were observed in 18 of the intermediate- and high-risk patients, which are higher in percentage. This is similar to the results of the retrospective cohort by Bai et al. [20], in which high-risk patients had significantly elevated D-dimer levels. Silva et al. also reported abnormal levels of PT and D-dimers [21].

Coagulation tests PT and APTT were prolonged in 46 (74.19%) and 38 (61.29%) of the patients, respectively. Out of those, 46 patients had prolonged PT, and 32 (51.61%) patients presented with bleeding. Out of the 38 patients with prolonged APTT, 26 (41.93%) patients presented with bleeding. Another study carried out in Brazil revealed that 69% and 13% of the patients had prolonged PT and APTT, respectively [22]. In another study by Chang et al. in Taiwan, it was found that patients who presented with prolonged PT and APTT had an increased risk for bleeding and were directly associated with significant thrombo-hemorrhagic complications [23].

In our study, a greater number of patients presented with bleeding whose platelet count was <50 × 10^9^/L. There were marked differences in platelet count for patients between the risk groups. Patients in the intermediate- and high-risk groups presented with lower platelet counts, whereas patients in the low-risk group presented with relatively increased platelet counts. The total WBC count was higher in patients designated as high-risk as compared to the low- or intermediate-risk groups. The P-value for both platelet count and WBC count was less than 0.05, indicating a statistically significant association. These results substantiate the results reported by Song et al. in China, in which they found similar WBC count profiles for the high-risk group [24]. Contrary to our study, they did not find significant differences in the platelet counts between the low- and intermediate-/high-risk groups. However, low counts were associated with bleeding similar to the results of our study. In the study by Naymagon et al., patients who presented with a higher WBC count were more prone to have bleeding [25]. However, in our study, a higher number of patients presented with bleeding who had a relatively lower WBC count of <10 × 10^9^/L than patients who had a higher WBC count of >10 × 10^9^/L and presented with bleeding.

Fibrinogen levels showed variable patterns in our study. Overall, more patients were seen with normal levels of fibrinogen across all three risk groups as compared to the low- or high-risk groups. Only 16 out of the 62 patients had low fibrinogen at presentation. The bleeding status was also different for fibrinogen levels with the highest number of 24 of the patients who presented with bleeding who had normal fibrinogen levels. This was followed by a low fibrinogen level group with 12 patients presenting with bleeding. These results contrasted with the studies conducted by Chu et al. [26], Kim et al. [27] in China, and Lee et al. [28] in Korea, in which low fibrinogen levels were associated with a higher risk or incidence of bleeding at presentation.

A study from Pakistan by Altaf et al. [29] reported similar results to our study, in which a greater number of patients had a high-risk score and reported bleeding in 53.5% of patients. They observed the mean hemoglobin, total leukocyte count, and platelet count as 8.3 g/dL, 39.6 × 10^9^/L, and 40 × 10^9^/L, respectively. Karim et al. conducted a retrospective study on 26 patients, in which 13 (50%) of the patients were at high risk [30]. The mean values for platelet count, D-dimer, fibrinogen, WBC count, and hemoglobin were 40 × 10^9^/L, 13.1 mg/L, 86 mg/dL, 28.1 × 10^9^/L, and 8.5 g/dL, respectively.

Limitations

This was a single-center study. The study design was an observational study design, and the sample size was small. In the future, prospective, multicenter, national collaborative studies are needed to be done in Pakistan. Multivariate analysis was not also performed due to the small sample size and missing follow-up data.

## Conclusions

The results of our study suggest that regardless of bleeding symptoms, coagulation parameters must be investigated at the time of diagnosis in patients with suspected acute promyelocytic leukemia, and in addition to ATRA, transfusion of fresh frozen plasma should be done. It has clinical value, and adding it to the algorithm of treatment would be beneficial to the patients in the developing world, where resources are already meager. Longitudinal studies with a large cohort in the future are needed in this context.
